# Efficacy of top flat magnetic stimulation for chronic pelvic pain in men: preliminary results

**DOI:** 10.1038/s41443-023-00822-1

**Published:** 2024-01-18

**Authors:** Nicola Mondaini, Mauro Gacci, Tommaso Cai, Francesco Lotti, Vincenzo Li Marzi, Fabio Crocerossa, Andrea Abramo, Francesco Cantiello, Irene Fusco, Alessandra Comito, Sara Tanguenza, Rocco Damiano

**Affiliations:** 1 Department of Urology, Magna Graecia University of Catanzaro, Catanzaro, Italy; 2 Department of Minimally Invasive and Robotic Urologic Surgery and Kidney Transplantation, University of Florence, Florence, Italy; 3 Department of Urology, Santa Chiara Regional Hospital, Trento, Italy; 4 Sexual Medicine and Andrology Unit, Department of Experimental and Clinical Biomedical Sciences, University of Florence, Florence, Italy; 5 El.En. Group, 50041 Calenzano, Italy; 6 Pelvic Pain Centre-Florence, Florence, Italy

**Keywords:** Medical research, Preclinical research

## To the Editor

The syndrome of chronic prostatitis/chronic pelvic pain syndrome (CP/CPPS) may include pelvic/ genital pain, ejaculatory pain, anxiety, depression and erectile dysfunction [1] which severely affects people’s quality of life.

Management of CP/CPPS remains a huge challenge for care providers and a major burden for healthcare systems. There are currently many approaches to its management, using both pharmacological and non‐pharmacological interventions [2]. Pharmacological interventions include the use of Alpha‐blockers, 5‐alpha reductase inhibitors, antibiotic therapy (quinolones, tetracyclines and other agents), analgesics (non‐steroidal anti‐inflammatory drugs (NSAIDs), pregabalin), phytotherapy (pollen extract and bioflavonoids), Botulinum toxin A, Allopurinol and traditional medicine (traditional Chinese medicine, etc.). Among non‐pharmacological interventions there are acupuncture and electroacupuncture, local thermotherapy, extracorporeal shockwave therapy, electromagnetic chair, myofascial trigger point release, biofeedback, circumcision, physical activity, psychological support, prostatic surgery and electrical stimulation. Particularly, electrical stimulation involves passing a small electrical current through the muscles around the bladder. Making them contract can help improve the patient’s pelvic floor muscle tone.

Among these, pelvic floor electromagnetic/magnetic therapy can represent a non-invasive option for CP/CPPS in men, with a neuromodulating effect on pelvic floor spasms and neural hypersensitivity, as already demonstrated in the literature [3].

On these bases this observational study evaluates the efficacy and the safety of a new device that uses Top flat Magnetic Stimulation (DEKA M.E.L.A. Calenzano, Italy) for the treatment of hypertonia in men affected by CP/CPPS condition.

DR ARNOLD (DEKA M.E.L.A. Calenzano, Italy) consists of a main unit and a chair applicator (see Supplementary Fig. 1), and the stimulation is generated by electromagnetic fields with a homogenous profile (TOP FMS—TOP Flat Magnetic Stimulation). This homogeneity of magnetic field distribution (see Supplementary Fig. 2) allows greater recruitment of muscle fibers without creating any areas of uneven stimulation intensity.

All patients underwent 8 treatments with the DR ARNOLD System (DEKA M.E.L.A. Calenzano, Italy). Sessions were conducted twice weekly for four consecutive weeks. For all patients enrolled in this study, the Overtone/Pain protocol (muscle work aimed at muscle inhibition and reduction of pain (hyperactivity and hypertone)) after the first two minutes of a warm-up phase, was selected. Overtone/Pain protocol consists of a warm-up and muscle relaxation phase. The protocol uses low frequencies (around 10 Hz) in a trapezoidal shape, creating a homogeneous distribution of the electromagnetic field that does not generate regions of different stimulation intensities avoiding an overstimulation of the area. Each session lasted 29 min with a medium intensity of 35% (range of 30–55%). Data were collected at baseline and 1-month follow-up (1MFU) after the last treatment session.

The study population consisted of 20 male patients affected by CP/CPPS (persistent or recurrent symptoms and no other urogenital pathology for ≥3 of the previous 6 months), with a mean age of 33.4 ± 11.9 years old (ranging from 18-55).

Exclusion criteria were: patients with genital infections, malignant tumors, severe neurological diseases, obesity and persons with pacemakers or metal implants.

The diagnosis of CP/CPPS was performed in all patients by an experienced urologist with a physical examination, including assessment of external genitalia, pelvic floor muscle dysfunction and discomfort or pain perceived by patients.

The Italian version of The National Institute of Health ‐ Chronic Prostatitis Symptom Index (NIH‐CPSI) (evaluating ‘pain’, ‘urinary symptoms’ and ‘impact on quality of life’), which is a validated measure commonly used to measure CP/CPPS symptoms [4], was administered to all patients. The mean ‘pain’ score, ‘urinary symptoms’ and patient’s quality of life (‘QL’) were evaluated at baseline and 1MFU after the last treatment session. The mean pain score was also assessed using the Pain Intensity Visual Analog Scale (VAS) at baseline and 1MFU after the last treatment.

The International Index of Erectile Function-5 (IIEF-5) [5] was provided before and 1MFU after the last treatment session. All possible side effects, including tendon pain, muscular pain, local erythema, skin redness and temporary muscle spasms, were evaluated during the entire treatment period. Informed consent was signed by the patients and archived.

**Fig. 1:**
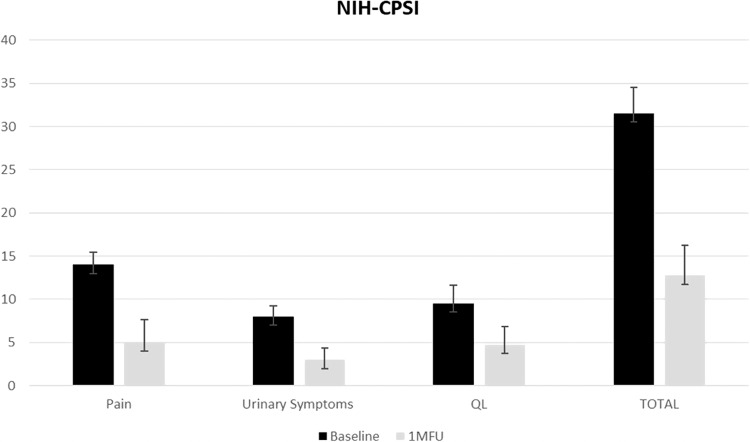
NIH-CPSI questionnaire mean results divided by different items (‘Pain’, ‘Urinary Symptoms’, ‘QL’) and total score at baseline and 1MFU after the last treatment session.

Changes in the total mean NIH‐CPSI score significantly decreased from 31.5 ± 2.9 at baseline to 12.75 ± 3.5 (*P* < 0.001) at 1MFU after the last treatment session showing an improvement in CP/CPPS symptoms (Fig. 1). The mean ‘pain’ score significantly decreased from 14 ± 1.5 at baseline to 5.0 ± 2.6 at 1MFU after the last treatment session (*p* < 0.01). The mean ‘urinary symptoms’ score significantly decreased from 8.0 ± 1.2 at baseline to 3.0 ± 1.4 at 1MFU after the last treatment session (*P* < 0.001). The mean ‘QL’ score significantly decreased from 9.5 ± 2.1 at baseline to 4.75 ± 2.1 at 1MFU after the last treatment session (*p* < 0.001).

The total mean IIEF-5 score significantly increased from 21.3 ± 2.7 at baseline to 24.3 ± 0.5 at 1MFU after the last treatment session (*p* < 0.001), showing better erectile functioning.

Finally, the mean VAS score significantly decreased from 7.05 ± 1.0 at baseline to 3.0 ± 0.9 at 1MFU after the last treatment session (*p* < 0.001).

All patients completed the planned treatment and follow-up according to protocol. No patients experienced side effects or significant pain increase during or after treatment.

Our preliminary clinical findings clearly showed that TOP FMS improved CP/CPPS symptoms.

Furthermore, an improvement in the patient’s erectile functioning was demonstrated by the IIEF-5 results.

The TOP FMS appeared to induce an inhibitory effect on detrusor overactivity through both afferent and efferent stimulation of sacral nerve roots in a similar manner to electrical stimulation but with significant advantages. Patients have the freedom to remain dressed and seated comfortably thanks to the regular emission of the gradually supplied energy. For a precise position of the patient, the seat height has been adjusted, so that the patient’s legs are perpendicularly flexed, the thighs are parallel to the floor and the feet are flat on the ground. When subjects experience their muscles relaxing, they become more conscious and immediately resume their regular daily activities.

This technology could represent a new treatment option for CP/CPPS conditions.

Study limitations are represented by the small sample size and a lack of a control group.

Our long-term goal is to enroll more patients and to extend the follow-up period to further investigate this innovative and non-invasive therapy for the management of complicated pathologies such as CP/CPPS.

## Supplementary information

supplemenrtary Figure legends

Supplementary Figures

## Data Availability

Data are available from the corresponding author on reasonable request.
