# Supplementary material for: Efficacy of top flat magnetic stimulation for chronic pelvic pain in men: preliminary results
Source: Int J Impot Res. 2024 Jan 18;36(6):665–7. doi: 10.1038/s41443-023-00822-1 (PMC11377295; doi:10.1038/s41443-023-00822-1)
Supplement: Supplementary file 1 — supplemenrtary Figure legends [file 41443_2023_822_MOESM1_ESM.docx]

Supplementary Figure 1. Representation of DR ARNOLD’s chair. The device has a central unit and a chair applicator planned for deep pelvic floor area therapy. Courtesy of DEKA M.E.L.A company. (from published article, https://www.wjnu.org/index.php/wjnu/article/view/432/378)

Supplementary Figure 2. Magnetic field spatial profile, which allows the distribution of electromagnetic energy in a double-dome pattern. This mechanism allows the muscle to work at the same intensity in all treatable areas. Courtesy of DEKA M.E.L.A company (from published article, <https://www.ncbi.nlm.nih.gov/pmc/articles/PMC10224487/pdf/medicina-59-00879.pdf>).
