# Supplementary figures and images for: Efficacy of top flat magnetic stimulation for chronic pelvic pain in men: preliminary results
Source: Int J Impot Res. 2024 Jan 18;36(6):665–7. doi: 10.1038/s41443-023-00822-1 (PMC11377295; doi:10.1038/s41443-023-00822-1)

Supplementary Figure 1

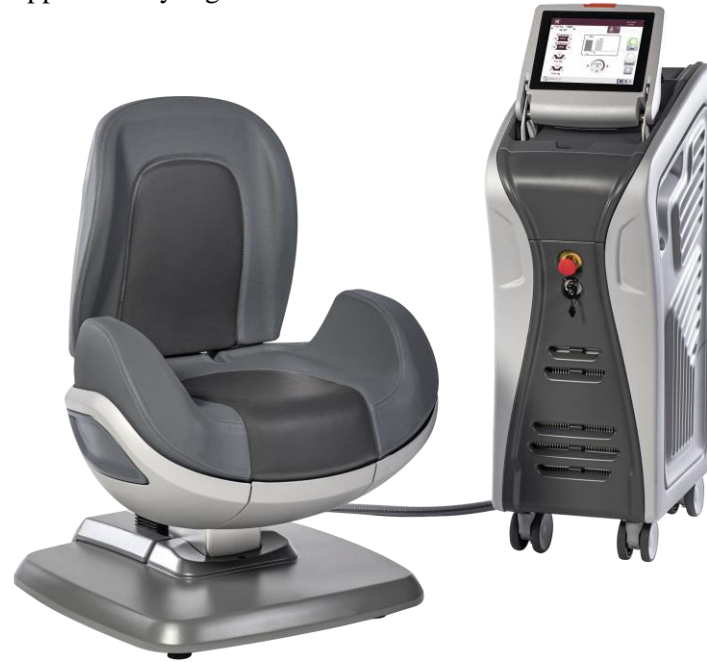

Supplementary Figure 2

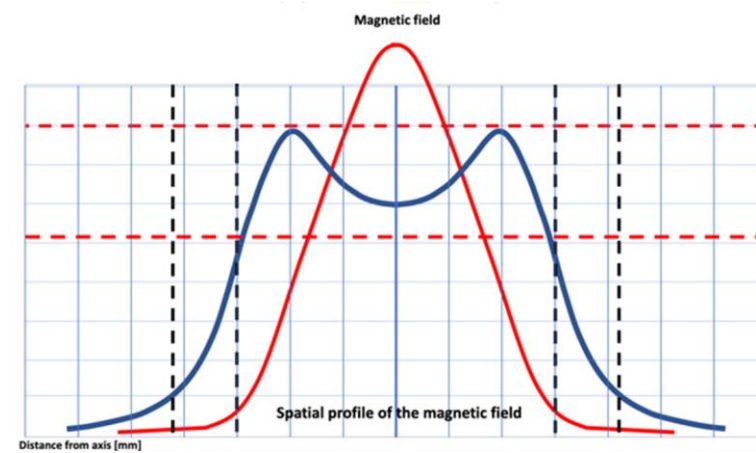

Supplement: Supplementary file 2 — Supplementary Figures [file 41443_2023_822_MOESM2_ESM.pdf]
